# Describing and Mapping the Research Trend of Scientific Publications on Arrhythmogenic Right Ventricular Cardiomyopathy Across Four Decades: A Bibliometric Analysis

**DOI:** 10.1002/clc.70051

**Published:** 2024-11-26

**Authors:** Leitong Mo, Ching‐Hui Sia, Weiqin Lin, Xifeng Zheng, Kaiyi Peng

**Affiliations:** ^1^ Department of Coronary Care Unit Maoming People's Hospital Maoming Guangdong China; ^2^ Department of Cardiology National University Heart Centre Singapore Singapore Singapore; ^3^ Department of Medicine Yong Loo Lin School of Medicine National University of Singapore Singapore Singapore; ^4^ Department of Internal Medicine Hospital of Guangdong University of Technology Guangzhou Guangdong China; ^5^ Department of Critical Care Medicine Maoming People's Hospital Maoming Guangdong China

**Keywords:** arrhythmogenic right ventricular cardiomyopathy, bibliometric analysis, diagnosis, fatal complications, molecular mechanism, prognosis, therapy

## Abstract

**Objectives:**

To perform a bibliometric analysis of publications of arrhythmogenic right ventricular cardiomyopathy (ARVC) from 1981 to 2023 to summarize the current publications and explore frontiers on this topic.

**Methods:**

We integrated the scientific publications on ARVC in the Web of Science (WOS) Core Collection database from January 1981 to September 2023, using the retrieval strategy of medical subject headings combined with keywords. We focused on articles and reviews that were published in English. Relevant information such as the journal and publisher, the title, authors, organizations, abstract, keywords, published date, and number of citations, were collected. Bibliometric analysis was performed and visualized by the R software and Microsoft Excel.

**Results:**

The results revealed a total of 4792 records related to ARVC from the WOS database, and 2992 original articles or reviews which were selected for bibliometric analysis. There were 79 countries and regions, 3724 research institutions, and 12 157 scholars who have published in this topic. The number of scientific publications of ARVC increased year‐by‐year, with an annual growth rate of 12.12%. We also investigated the top 10 contributing countries, organizations with affiliations, most influential researchers, highest‐cited articles, and highest‐frequency keywords. In addition, the most active areas of research on ARVC included that of fatal complications, molecular pathological mechanisms, diagnosis, therapy, and prognosis respectively according to the keywords trend analysis.

**Conclusions:**

Our study reports the publication landscape of ARVC during the past four decades based on bibliometric analysis. This study provides a deeper understanding of the published literature on ARVC.

## Graphical abstract

Note: The graphical abstract of the study.

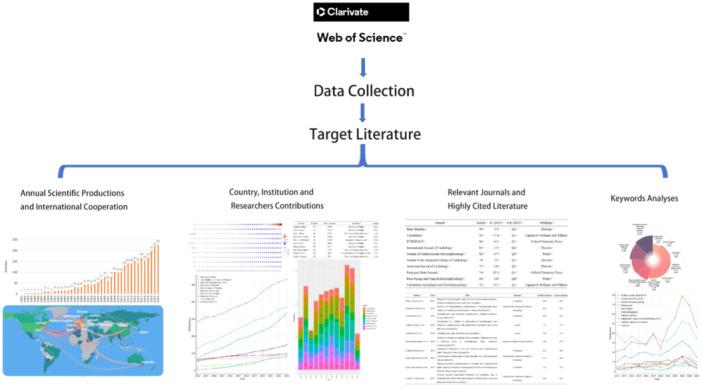

**Abbreviations**
ARVC: arrhythmogenic right ventricular cardiomyopathy
CMR: cardiac magnetic resonance
ICD: implantable cardioverter defibrillator
SCD: sudden cardiac death
WOS: Web of Science

## 1 Introduction

Arrhythmogenic right ventricular cardiomyopathy (ARVC), also known as arrhythmogenic right ventricular dysplasia, is a type of primary inherited cardiomyopathy whose hallmark is progressive replacement of right ventricular myocardium by fibrofatty tissue, accompanied by high risk of ventricular arrhythmias (VAs), sudden cardiac death (SCD), and heart failure (HF). In certain instances, the left ventricle may also be involved. ARVC was first reported by Dungan WT and colleagues who characterized ventricular tachycardia (VT) in children in 1981 [1], and subsequently by Marcus and colleagues who reported on 24 adults with similar clinical manifestations in 1982 [2]. According to estimates, the population prevalence of ARVC ranges from 1:5000 to 1:1000. Meanwhile, it is also recognized as a major cause of sudden cardiac arrest in young people and athletes [3]. Initially, ARVC was described as a congenital defect in the development of the right ventricular myocardium. The currently accepted pathogenetic mechanism involves genetic mutations encoding desmosomal‐related proteins as the leading cause of ARVC. Desmosomes belongs to an adhesion junction consisting of a symmetrical group of proteins that provide mechanical connections between cardiomyocytes. Genetic mutations of desmosomal‐related proteins, such as plakophilin‐2 (PKP2), desmoglein‐2 (DSG2), desmocollin‐2 (DSC2), plakoglobin (JUP) and desmoplakin (DSP), would disrupt the normal structural integrity of cardiomyocytes, thereby promoting the gradual replacement of the myocardium by fibro‐fatty tissue, which ultimately results in structural and functional abnormalities of the myocardium [4]. The pathological process commonly involves multiple complex molecular biological mechanisms, such as inflammation, canonical and non‐canonical WNT signaling, the Hippo–Yes‐associated protein (YAP) pathway and transforming growth factor‐β signaling [5].

There has been substantial progress in understanding of the pathogenesis, diagnosis, treatment, and long‐term prognosis of ARVC across the past four decades. Moreover, with an increase in the number of scientific publications, it is necessary to comprehensively review the literature in this field. Bibliometric analysis refers to the quantitative analysis of published academic literature using mathematical and statistical methods to investigate the development of a certain topic within a specific time frame. This method emphasizes the impact of publications; the contributions of countries and regions, organizations, individuals, and as well as the potential research for the future [6]. To the best of our knowledge, there is yet to be a bibliometric analysis performed on the topic of ARVC. Thus, we conducted such an analysis to summarize the current publications and explore potential frontiers on this topic.

## 2 Materials and Methods

### 2.1 Literature Source and Retrieval Strategy

The Web of Science (WOS) is recognized as a high‐impact, multidisciplinary academic database and widely used for bibliometric analysis [7, 8]. We retrieved the scientific publications included in the WOS Core Collection database from January 1981 to September 2023, with a search strategy as the medical subject headings combine with the text words. The medical subject headings and the text words were originated from the annotation of National Library of Medicine database (https://www.ncbi.nlm.nih.gov/). The detailed search strategy was displayed as follows: (((((((TS = (Arrhythmogenic Right Ventricular Dysplasia)) OR TS = (ARVC)) OR TS = (ARVC‐Dysplasia)) OR TS = (ARVC Dysplasia)) OR TS = (Ventricular Dysplasia, Right, Arrhythmogenic)) OR TS = (ARVD‐C)) OR TS = (Right Ventricular Dysplasia, Arrhythmogenic)) OR TS = (Arrhythmogenic Right Ventricular Dysplasia‐Cardiomyopathy) OR TS = (ARVC) and Article or Review Article (Document Types) and English (Languages). Literature retrieval was conducted independently by two authors (Xifeng Zheng and Ching‐Hui Sia). Detailed information on the identified publications, such as the journal and publisher, the title, authors, organizations, abstract, keywords, published date, and number of citations, were saved in a “.biblix” format. This study followed the Strengthening the Reporting of Observational Studies in Epidemiology (STROBE) reporting guidelines [9].

### 2.2 Bibliometric Analysis

**Figure 1:**
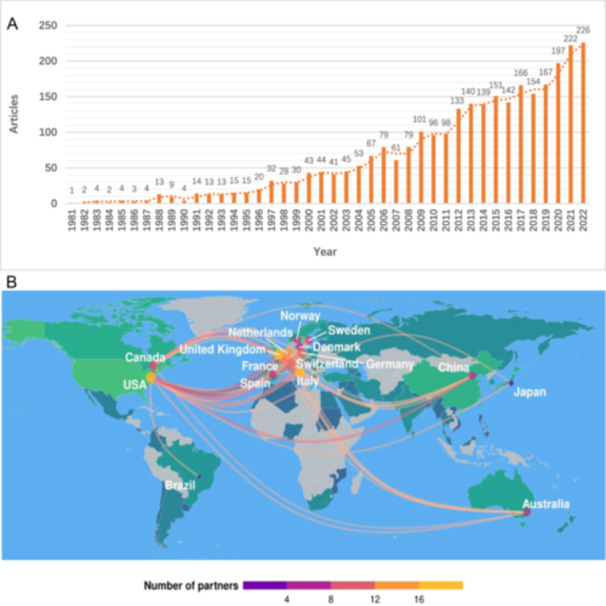
(A) Global annual scientific productions of ARVC since 1981. The horizontal axis represents time (years), and the vertical axis represents the global number of articles on ARVC published each year. (B) Distribution of major international collaborations. Major international ARVC research collaborations are marked on the map.

We analyzed the academic publications of ARVC since 1981. The annual growth rate of publications reflects the interest in the topic. We also explored the countries and regions, organizations, and scholars who took part in ARVC research in the past four decades, and report the top 10 contributing countries, organizations and their affiliations, and the most influential researchers. Moreover, journals with most ARVC‐related publications, and literature with the most citations involving ARVC were also analyzed. Finally, we investigated the highest‐frequency keywords by keyword trend analysis to explore the trends in this field [10]. The bibliometric analysis was processed and visualized by R software (version 4.0.3) with the “bibliometrix” program (version 4.0) [11] and Microsoft Excel 2016. The flowchart of the research is illustrated in Supporting Information S1: Figure 1.

## 3 Results

### 3.1 Overview of the Global Publications

There was a total of 4792 records related to ARVC from the WOS database, and 2992 publications matched the search strategy and was selected for further analysis. In summary, during the past four decades, there were 79 countries and regions, about 3724 research organizations with affiliations, and nearly 12 157 scholars joined the field. The number of scientific achievements kept rising year‐on‐year, with an annual growth rate of 12.12% (Figure 1A). Major international collaborations involved Australia, Brazil, Canada, China, Denmark, France, Germany, Italy, Japan, the Netherlands, Norway, Spain, Sweden, Switzerland, United Kingdom, and United States of America (Figure 1B, sorted by country in alphabetical order).

### 3.2 The Top 10 Contributing Countries, Organizations, and Researchers of ARVC

**Figure 2:**
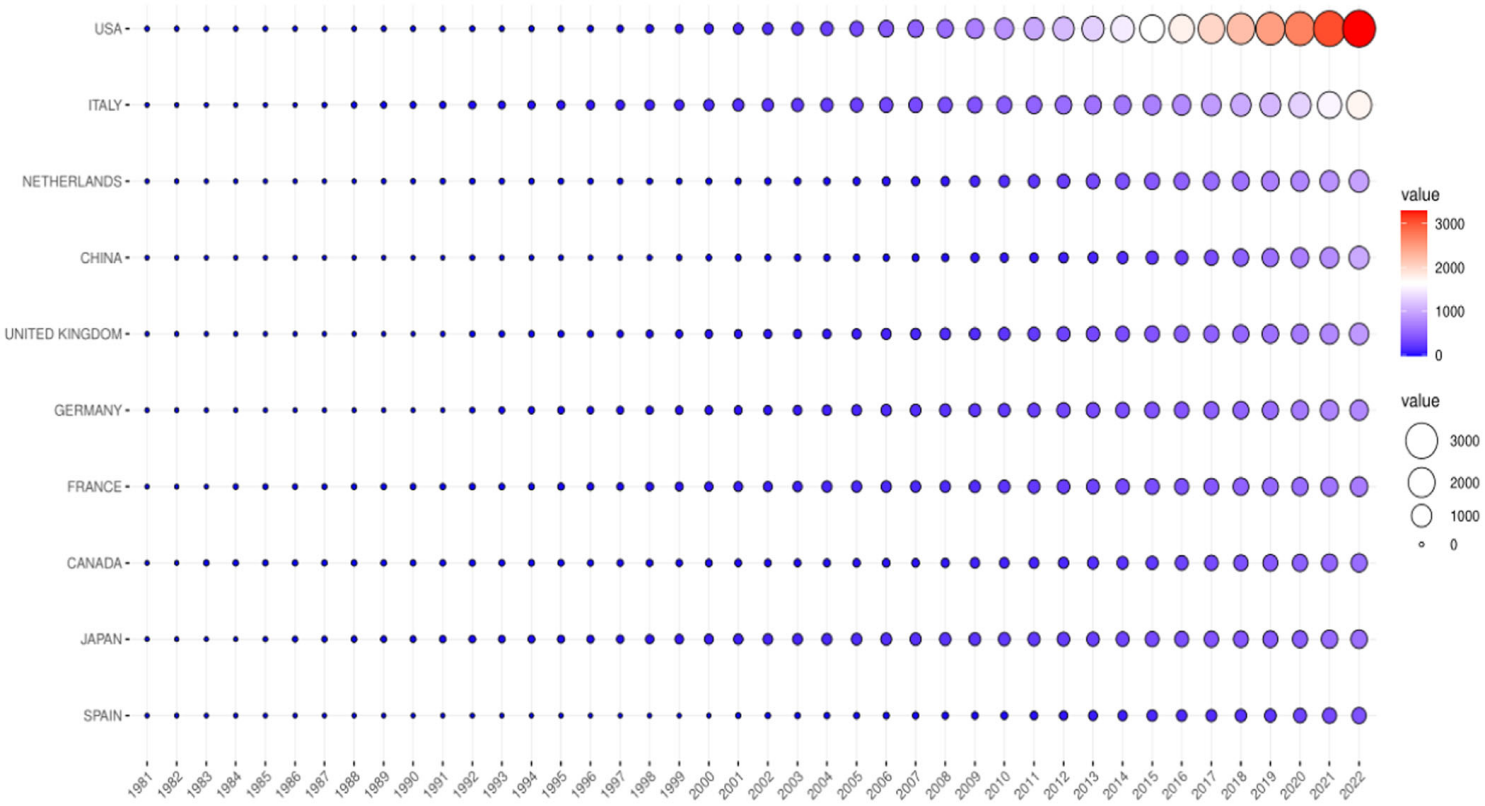
Scientific publication from the top 10 contributing countries over time. The horizontal axis represents time (years). The vertical axis represents the top 10 countries with their cumulative scientific publications and related value over time (1981–2022).

**Figure 3:**
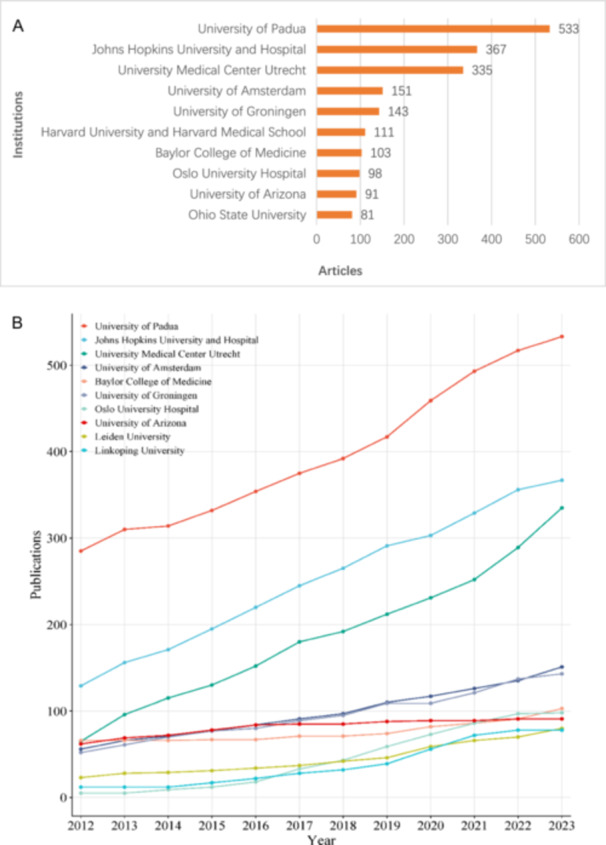
(A) The top 10 institutions with most scientific productions accumulated. The horizontal axis represents the total number of scientific productions since 1981, and the vertical axis represents the top 10 organizations with affiliations with the most accumulated scientific publications from 1981 to 2022. (B) The top 10 institutions with most scientific publications on ARVC since 2012.

Globally, the United States of America contributed the most scientific publications on ARVC, followed by Italy, the Netherlands, China, United Kingdom, Germany, France, Canada, Japan, and Spain (Figure 2). The top 10 research organizations with the most publications are, in descending order of the number of publications, the University of Padua, Johns Hopkins University and Hospital, University Medical Center Utrecht, University of Amsterdam, University of Groningen, Harvard University and Harvard Medical School, Baylor College of Medicine, Oslo University Hospital, University of Arizona, and Ohio State University (Figure 3a). Moreover, we have depicted the publication trend of various institutions over the past decade (Figure 3b) The H‐index is an indicator which reflects a scholar's academic achievement. It combines two key metrics, the number of publications and citation counts, and is defined as the number of publications with corresponding citation counts ≥ H [12]. Utilizing the H‐index and publications over the past decade as evaluation criteria (Supporting Information S1: Table 1 and Supporting Information S1: Figure 2), the most influential scholar of ARVC is Gaetano Thiene from University of Padua, Italy, with the highest score of 62. This is followed by Cristina Basso (University of Padua), Hugh Calkins (Johns Hopkins University), Domenico Corrado (University of Padua), William J McKenna (University College London), Barbara Bauce (University of Padua), Andrea Nava (University of Padua), Harikrishna Tandri (Medical University of South Carolina), Daniel P Judge (Medical University of South Carolina), and Cynthia A James (Johns Hopkins University).

**Figure 4:**
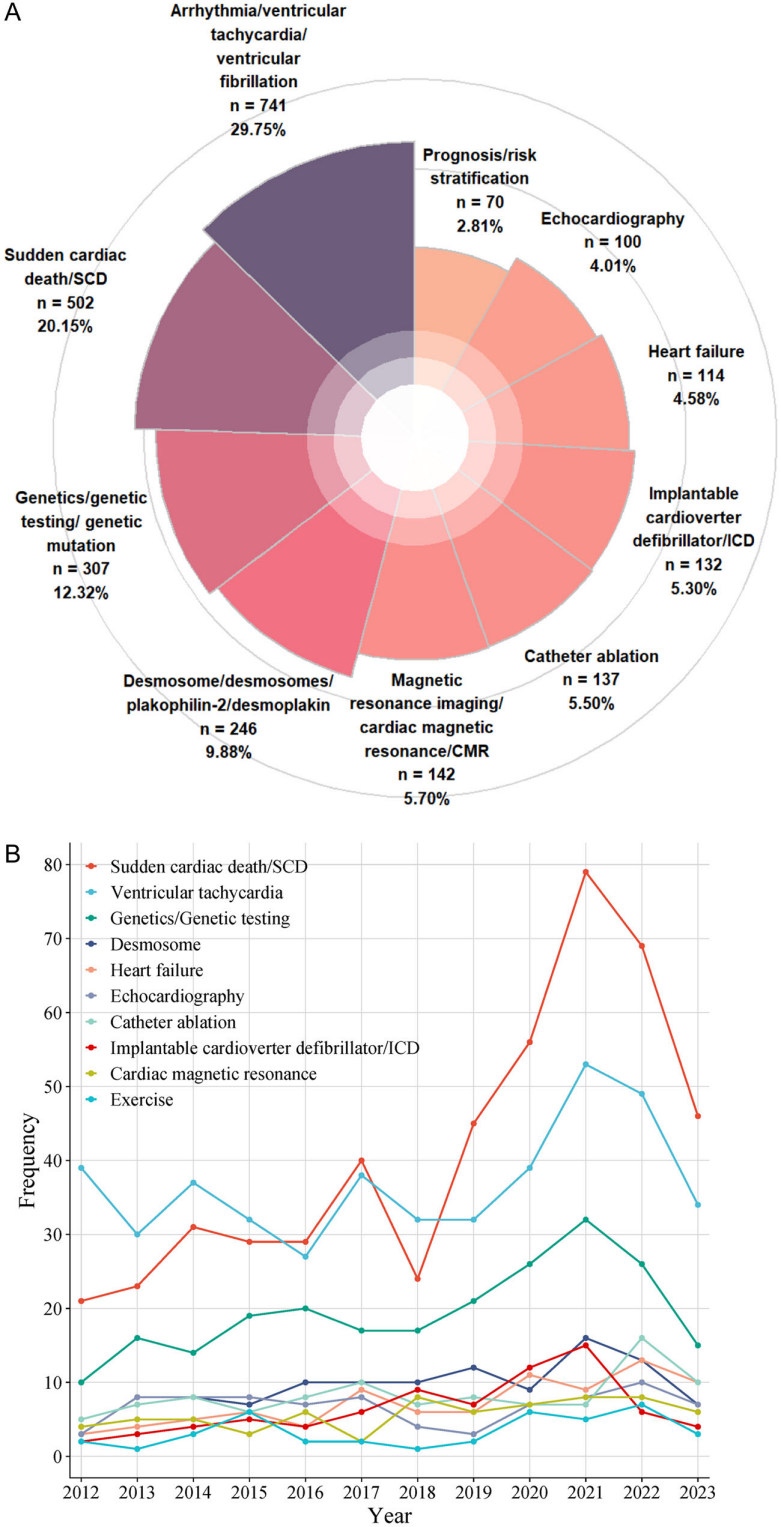
(A) Top 10 highest‐frequency keywords about ARVC. The figure concluded the top 10 highest‐frequency keywords and order by the percentages. (B) The keywords frequency trend since 2012.

### 3.3 Influential Journals and Highly‐Cited Articles of ARVC

The top 10 journals with the most academic publications of ARVC are, in descending order of number of publications, the Heart Rhythm, Circulation, EUROPACE, International Journal of Cardiology, Journal of Cardiovascular Electrophysiology, Journal of the American College of Cardiology, American Journal of Cardiology, European Heart Journal, Pace‐Pacing and Clinical Electrophysiology and Circulation‐Arrhythmia and Electrophysiology (Supporting Information S1: Table 2). The top 10 highly‐cited literature during the past four decades are listed in Table 1, most of which belonged to guidelines or clinical trials. They were concentrated in influential journals such as Circulation, Journal of the American College of Cardiology, and the Lancet. In summary, the highly cited literature contributes in following key ways: Firstly, they have proposed or refined diagnostic criteria, significantly impacting the standardization of patient diagnosis and care. Secondly, the identification of specific genetic mutations associated with ARVC has enhanced our understanding of disease pathogenesis and integrated genetic testing into the diagnostic process. Lastly, they provide evidence to inform treatment guidelines and improve patient management.

**Table 1:** Top 10 most cited papers on ARVC in the past four decades.

Authors	Year	Title	Journal	Global citations	Local citations
Frank I. Marcus et al.	2010	Diagnosis of arrhythmogenic right ventricular cardiomyopathy/dysplasia: Proposed Modification of the Task Force Criteria [13]	Circulation	908	487
Domenico Corrado et al.	1997	Spectrum of Clinicopathologic Manifestations of Arrhythmogenic Right Ventricular Cardiomyopathy/Dysplasia: A Multicenter Study [14]	Journal of the American College of Cardiology	664	447
Cristina Basso et al.	1996	Arrhythmogenic right ventricular cardiomyopathy. Dysplasia, dystrophy, or myocarditis? [15]	Circulation	609	431
Godfrina McKoy et al.	2000	Identification of a deletion in plakoglobin in arrhythmogenic right ventricular cardiomyopathy with palmoplantar keratoderma and woolly hair (Naxos disease) [16]	Lancet	753	372
Cristina Basso et al.	2009	Arrhythmogenic right ventricular cardiomyopathy [3]	Lancet	558	372
Alessandra Rampazzo et al.	2002	Mutation in Human Desmoplakin Domain Binding to Plakoglobin Causes a Dominant Form of Arrhythmogenic Right Ventricular Cardiomyopathy [17]	American Journal of Human Genetics	439	272
Kalliopi Pilichou et al.	2006	Mutations in Desmoglein‐2 Gene Are Associated with Arrhythmogenic Right Ventricular Cardiomyopathy [18]	Circulation	402	260
Srijita Sen‐Chowdhry et al.	2008	Left‐Dominant Arrhythmogenic Cardiomyopathy: An Under‐Recognized Clinical Entity [19]	Journal of the American College of Cardiology	437	260
Srijita Sen‐Chowdhry et al.	2007	Clinical and Genetic Characterization of Families with Arrhythmogenic Right Ventricular Dysplasia/Cardiomyopathy Provides Novel Insights Into Patterns of Disease Expression [20]	Circulation	363	255
Cynthia A. James et al.	2013	Exercise Increases Age‐Related Penetrance and Arrhythmic Risk in Arrhythmogenic Right Ventricular Dysplasia/Cardiomyopathy–Associated Desmosomal Mutation Carriers [21]	Journal of the American College of Cardiology	436	245

### 3.4 Keywords and Hotspots Trend Analyses

A total of 3565 keywords were noted on the keywords section among the 2992 selected articles. We focused on the top 100 keywords according to the frequency and selected the top 10 keywords based on similar implications. In order of the proportion of keywords, they were arrhythmia/VT/ventricular fibrillation (VF), SCD, genetics testing/genetic mutation, desmosome/PKP2, cardiac magnetic resonance (CMR), catheter ablation, implantable cardioverter defibrillator (ICD), HF, echocardiography, prognosis, and risk stratification respectively (Figure 4a). We also present the trend of keywords over the past decade (Figure 4b). In addition, these keywords can be summarized as four major research topics including (1) Fatal clinical complications, (2) Molecular pathogenesis mechanism, (3) Diagnosis and therapy, and (4) Risk stratification and prognosis, respectively. They represent the key thrusts of ARVC research in the past four decades and may direct the future research direction of this topic.

## 4 Discussion

### 4.1 Overview

The present study provides an overview of the publication landscape of ARVC over the past four decades using bibliometric analysis. The results demonstrate a continuous increase in the number of academic publications on ARVC, indicating a sustained interest among cardiologists worldwide. Since the first seminal literature on ARVC was reported by Dungan WT and colleagues in the American Heart Journal in 1981, the United States has been the foremost contributor to scientific publications on ARVC and has advocated for international collaboration in this field on a global scale. These collaborative networks enable smooth and efficient sharing of knowledge, resources, and data, ultimately accelerating scientific progress in this domain. European countries, particularly Italy and Germany, exhibit a higher incidence of genetic mutations linked to ARVC, prompting intensified research to elucidate its underlying mechanisms and develop effective therapeutic strategies. Moreover, both the United States and European countries prominence in ARVC research arises from their robust research infrastructures, which encompass elite universities and medical centers, a deep‐rooted tradition in cardiovascular research, significant funding, and a vast patient population conducive to clinical trials. Notably, the University of Padua in Italy stands out as an institution that has gathered influential scholars (i.e., Gaetano Thiene, Cristina Basso, Domenico Corrado, Andrea Nava, and Barbara Bauce) and achieved significant academic accomplishments. It is worth mentioning that the University of Padua played a dominant role in shaping the latest modified diagnostic criteria for ARVC [22], leading to a significant advancement in our understanding of the disease. The keywords trend analyses of the publications can be summarized as four major research topics including (1) Fatal clinical complications, (2) Molecular pathogenesis mechanism, (3) Diagnosis and therapy, and (4) Risk stratification and prognosis, respectively, which reflect the research hotspots evolution of ARVC. Consequently, we aim to review the achievements and delve into the frontiers of these key research hotspots.

### 4.2 Molecular Pathogenesis Mechanism and Genetic Testing

ARVC is primarily caused by mutations in desmosomal‐related genes [23] and is predominantly inherited in an autosomal pattern with variable penetrance and expressivity. The desmosome consists of three principal components: (1) DSP, which binds to intermediate filaments (i.e., cardiac desmin); (2) transmembrane proteins (i.e., desmosomal cadherins), including DSC2 and DSG2; (3) linker proteins (i.e., proteins of the armadillo family), including JUP and PKP2, which mediate interactions between the desmosomal cadherin tails and DSP [24]. To date, at least 13 genes (JUP, DSP, PKP2, DSG2, DSC2, CTNNA3, TMEM43, DES, TTN, LMNA, PLN, TGFB3, and RYR2) have been identified as responsible for approximately 60% of all ARVC cases [25]. Six of which (PKP2, DSP, DSC2, DSG2, JUP, and TMEM43) had solid evidence and were judged to be definitive for ARVC [26].

The DSP gene was the first gene associated with the classical autosomal dominant form of ARVC [17]. It is a crucial desmosomal protein essential for cardiac force transmission, serves as a linker protein, connecting the desmosome to a cytoskeletal protein network, which encompasses intermediate filament proteins. The desmosome and its constituent proteins regulate intercellular communication. Alessio Gasperetti et al. conducted a study involving 252 ARVC patients with DSP gene variation, enrolled from 20 institutions spanning three continents. The study reported that 37.3% of these patients experienced VAs over a follow‐up period of 44.5 months (IQR: 19.6−78.3). Meanwhile, neither age nor male sex were significantly associated with the occurrence of VAs [27]. Similarly, Reza et al. reported 19 individuals diagnosed with ARVC due to variants in DSP, approximately 40% exhibited left ventricular enlargement during the initial assessment. Malignant arrhythmias were prevalent in this cohort, accounting for 42% of cases. Additionally, a substantial portion of the cohort underwent primary (68%) and secondary prevention ICD implantation, with a minority receiving ablation for VAs (16%) [28]. Moreover, recessive mutations in DSP have been observed in Carvajal syndrome, a condition characterized by palmoplantar keratoderma, woolly hair, and biventricular dilated cardiomyopathy [29].

The PKP2 gene is considered the most common genetic mutation in ARVC, accounting for approximately 20%−46% of cases. The protein encoded by the PKP2 gene plays an essential role in linking JUP and DSP at the intercalated discs. The malfunction of the PKP2 protein causes a chain of events that may lead to the disruption of intracellular signaling, of electrophysiology and of the cardiac transcription program [30]. A recent study reported genetic findings from a large transatlantic cohort, which revealed that 463 of 577 participants (account for 80%) had a single PKP2 mutation, with fewer heterozygous carriers of mutations in other ARVC‐associated genes [31]. Accumulating clinical research suggests that mutations in the PKP2 gene are often associated with malignant arrhythmic. In the Nordic ARVC registry multinational cohort with 419 patients, with a mean of 11.2 ± 7.4 years long‐term follow‐up, Christensen et al. demonstrated PKP2 genotype to be more arrhythmic than DSC2/DSG2/DSP or gene‐negative carrier status (HR = 0.63, 95% CI = 0.42−0.95) [32].

Advances in understanding molecular genetic mechanisms and genetic sequencing techniques have propelled the integration of genetic testing as a fundamental diagnostic criterion for ARVC since 2010 [13], particularly among patients with a familial history. The expert consensus statement issued in 2022 recommends comprehensive genetic testing for all patients exhibiting consistent phenotypic features of ARVC, regardless of familial history, including post‐mortem cases. It is advisable to conduct genetic testing for first‐tier definitive disease‐associated genes, including such as PKP2, DSP, DSG2, DSC2, JUP, TMEM43, PLN, FLNC, DES, and LMNA. Meanwhile, once the disease‐causative variant has been identified, variant‐specific genetic testing is recommended for family members and related relatives [33]. Genetic testing also holds significant value in assessing the prognosis of ARVC patients. Patients carrying two pathogenic variants tend to experience major adverse cardiovascular events earlier than those with one or no remaining reclassified variants. Specifically, the hazard ratios for these patients are 1.9 (95% CI, 1.2−2.9) and 1.8 (95% CI, 1.1−2.8), respectively [34]. Additionally, it is crucial to interpret genotyping results with thoroughness and caution to reduce the potential for misdiagnosis.

### 4.3 Cardiac Imaging

Cardiac imaging such as echocardiography and CMR, is essential to diagnosis and risk stratification of ARVC in evaluating morpho‐functional and myocardial structural abnormalities aspect. Echocardiography is a convenient non‐invasive test that provides an overall assessment of ventricular function and detects wall motion abnormalities. However, it has limitations in imaging the right ventricle and does not provide myocardial tissue characterization. Therefore, it is often supplemented by CMR, which offers high‐spatial‐resolution tomographic evaluation of the heart. CMR accurately assesses ventricular volumes, identifies segmental kinetic anomalies, and detects tissue alterations, including fibrosis distribution, myocardial edema, and fatty substitution [35]. The late gadolinium‐enhanced (LGE) technique of CMR not only visualizes the hallmark lesions of ARVC (fibrofatty replacement of the right ventricular myocardium) but also predicts arrhythmic events [36]. Consequently, CMR has been recommended in the 2020 Padua Criteria and the latest 2023 European Task Force consensus report for ARVC diagnosis [22, 37].

### 4.4 Clinical Treatment and Prevention of Fatal Complications

The fundamental management strategy for ARVC aims to reduce the risk of SCD and improve the quality of life by alleviating arrhythmic and HF symptoms. ICD placement is a well‐established treatment to prevent SCD, particularly in patients with a history of ventricular arrythmia. However, the necessity of ICD therapy for primary prevention in patients without a history of sustained VA remains a subject of further investigation due to concerns about inappropriate shocks and other disadvantages [38]. Risk factors for SCD in ARVC populations encompass male gender, syncope, both sustained and non‐sustained VAs, T‐wave inversion in inferior leads, dysfunction of both right and left ventricles, a complex genotype, and family proband status [39]. Based on the identified risk factors, The researchers have developed a prediction model, following ARVC patients longitudinally across 14 academic centers in six countries, which has undergone external validation to precisely stratify risk and aid in decision‐making regarding ICD therapy for the prevention of SCD in ARVC patients [40, 41]. Notably, based on the keyword trend analysis, “exercise” emerged as one of the popular keywords in the past decade. Exercise restriction plays a pivotal factor in preventing SCD, particularly among patients harboring mutations in desmosomal‐related genes. Competitive or high‐intensity leisure sports have been shown to enhance penetrance, promote the incidence of VAs and expedite the progression to ventricular dysfunction among carriers of pathogenic desmosomal variants [21]. The European Society of Cardiology guidelines recommend a maximum of 150 min of low to moderate‐intensity exercise per week for affected and at‐risk individuals [42]. Additionally, it is essential to minimize the frequency of ICD therapies through a combination of antiarrhythmic pharmacologic therapy and maintaining electrolyte balance. In recent years, catheter ablation has emerged as an alternative treatment for ARVC patients with frequent VT. The therapeutic effect has significantly improved with the combination of endocardial and epicardial ablation for VT [43].

HF is a late clinical manifestation of ARVC that significantly affects the quality of life. Due to the predominantly right heart phenotype and the absence of left heart‐associated symptoms, a considerable number of HF cases in ARVC patients may go unrecognized. A recent study focused on defining the prevalence of signs and symptoms of both left‐ and right‐sided HF in ARVC patients and found that 49% of them met the criteria for clinical HF [44]. Standard HF pharmacologic therapy, including angiotensin‐converting enzyme inhibitors, angiotensin II receptor blockers, beta‐blockers, and diuretics, can benefit these patients [39]. In cases of end‐stage HF with recurrent refractory VAs, heart transplantation may be considered as the ultimate solution [45]. However, the timing of evaluation, hemodynamic support options, and qualification for wait listing require careful consideration [46].

### 4.5 Development of Novel Medicines and Therapy

Advancements in basic science research have provided molecular insights into ARVC and hold the potential for developing new forms of treatment. For example, SB‐216763, an inhibitor of glycogen synthase kinase‐3β (GSK‐3β) and a pharmacological activator of the WNT/β‐catenin pathway, has been shown to suppress the ARVC phenotype in murine and zebrafish models [47, 48, 49]. It may represent a promising medical treatment for ARVC, but further evidence is needed to establish its clinical efficacy. Therefore, it is crucial to enhance collaboration among various national institutions to conduct multiple randomized controlled trials and develop and verify the efficacy of new drugs in the future.

The concept of “gene therapy” emerged in the 1970s, proposing that for specific indications, primarily monogenic diseases, the substitution of missing or mutated genes with normal alleles through gene addition could yield long‐term therapeutic benefits for affected patients, ultimately enhancing their quality of life [50, 51]. Currently, three types of gene therapy are available: gene replacement therapy, gene silencing therapy, and direct genome editing. In gene replacement therapy, a wild‐type gene is expressed using a promoter within a viral vector to restore function in a loss‐of‐function genetic variant. Gene silencing therapy is typically used to downregulate the expression of mutant genes, specifically allele‐specific silencing, and is primarily applied to missense variants that cause alterations in protein function, such as single‐nucleotide substitutions. In direct genome editing, CRISPR‐Cas9 technology is used to precisely target and cleave DNA at a specific location in the genome, determined by the unique sequence of a guide RNA [52]. Several ongoing preclinical studies targeting pathogenic variants in the PKP2, PLN, and DSG2 genes have observed promising outcomes, signifying a significant shift towards a new era in the treatment of cardiac genetic disorders. It is also necessary to acknowledge the challenges associated with translating these promising preclinical findings into clinically effective treatments. In the near future, initiating Phase 1 clinical trials with ARVC patients could provide valuable insights into their efficacy, safety, potential toxicity, and immunogenicity, thus furthering our understanding of specific disease characteristics [53].

### 4.6 Strengths and Limitations

Bibliometric analyses provide deeper insights into the evolution of research priorities and trends than traditional literature reviews, as they employ comprehensive and objective data analysis. The analysis exclusively relies on objective data, thereby eliminating biases that may arise from supervisory interventions. Additionally, it is worth noting the innovative aspect of our research. We incorporated medical subject headings and text words from PubMed, which are absent in WOS, to ensure a more comprehensive literature retrieval. However, several limitations should be acknowledged. Firstly, our study is limited to the WOS database, which may result in the exclusion of relevant studies. The exclusive use of WOS is justified despite potentially incomplete data, as alternative databases such as PubMed and Embase database lack full‐text access and the citation analysis required for comprehensive bibliometric assessments. Secondly, we focused specifically on English‐language original research articles and literature reviews, excluding other publication types such as conference papers, letters, and books. This approach guarantees the quality of the data for analysis, given that original research articles and literature reviews have undergone peer review before publication. However, it may introduce a selection bias. Lastly, bibliometric data evolves over time, especially considering our selected publication period spanning the past four decades. Recently published high‐quality literature may not have been frequently cited due to its recentness, potentially resulting in discrepancies between our research results and the actual situation. Despite these limitations having minor implications on the overall findings, they are not expected to significantly alter the principal trends identified in this research.

### 4.7 Prospect

Compared to other cardiomyopathy types, such as hypertrophic cardiomyopathy [54], ARVC demonstrates significant delays in annual scientific productivity, advancements in clinical diagnosis (particularly genetic testing), and therapeutic progress, including novel pharmacological and interventional strategies. The relatively low incidence of ARVC, combined with a lack of understanding and awareness regarding this disease, may account for these observed delays. Therefore, there is an urgent need to enhance both fundamental and clinical research efforts targeting ARVC, emphasizing its critical importance.

Future research and treatment of ARVC should prioritize the development of personalized medicine strategies, complemented by large‐scale, longitudinal studies to elucidate the natural progression of the disease. Additionally, exploring the potential of big data in ARVC management and fostering international collaborations for resource and data sharing is vital. Furthermore, the development and validation of biomarkers for early diagnosis, as well as the exploration of gene editing as a potential treatment, are imperative.

## 5 Conclusion

In summary, the present study employs bibliometric analysis to explore global research trends in ARVC spanning the past four decades, identifying key research hotspots in molecular pathogenesis, contemporary diagnostic criteria, and emerging therapeutic strategies. This study offers an in‐depth comprehension of the published literature pertaining to ARVC. As a complex inherited cardiomyopathy, ARVC has witnessed significant advancements in our comprehension of its genetic underpinnings. Significant advancements in clinical, genetic, and imaging research have enabled more precise diagnostic capabilities and broadened the therapeutic landscape for ARVC. With continued progress in this domain, the prospects for developing more personalized and efficacious therapeutic strategies for patients appear increasingly promising.

## Author Contributions

Leitong Mo was involved in statistics, software and article writing. Xifeng Zheng and Ching‐Hui Sia were responsible for study design, literature review. Weiqin Lin was responsible for writing review and English editing. Xifeng Zheng and Kaiyi Peng were responsible for scientific supervision. All authors reviewed and approved the final manuscript.

## Ethics Statement

The authors have nothing to report.

## Consent

The authors have nothing to report.

## Conflicts of Interest

The authors declare no conflicts of interest.

## Supporting information

Supporting information.

## Data Availability

The data sets utilized or analyzed in the present study are not publicly available in a repository. However, they can be accessed from the corresponding author upon reasonable request.
