# Supplementary material for: Describing and Mapping the Research Trend of Scientific Publications on Arrhythmogenic Right Ventricular Cardiomyopathy Across Four Decades: A Bibliometric Analysis
Source: Clin Cardiol. 2024 Nov 26;47(12):e70051. doi: 10.1002/clc.70051 (PMC11599429; doi:10.1002/clc.70051)
Supplement: Supplementary file 1 — Supporting information. [file CLC-47-e70051-s001.docx]

**Supplement Figure-1 The flowchart of the research process**


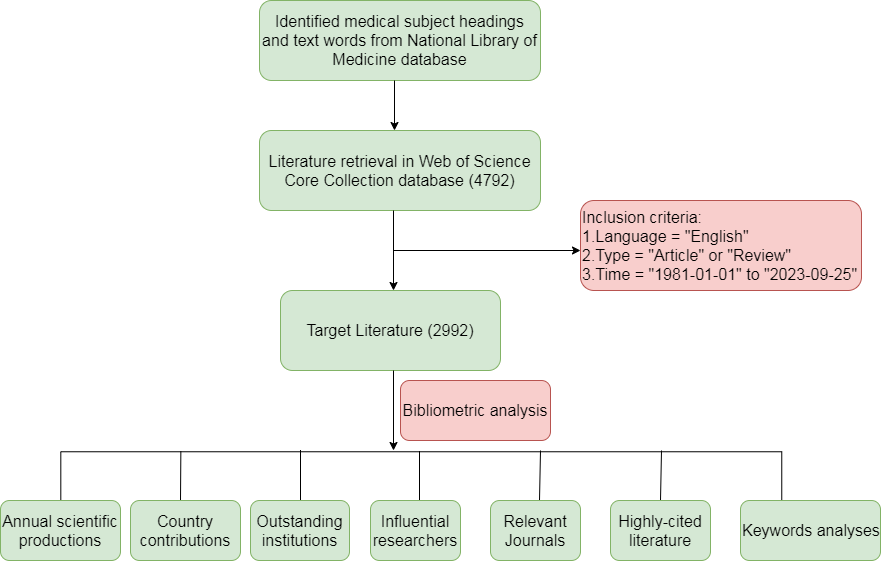


**Supplement table-1 The top 10 global most influential scholars on ARVC**

| Scholar | H-index | Total Citations | Institution | Country |
| --- | --- | --- | --- | --- |
| Gaetano Thiene | 62 | 17601 | University of Padua | Italy |
| Cristina Basso | 57 | 15167 | University of Padua | Italy |
| Hugh Calkins | 52 | 9778 | The Johns Hopkins University | USA |
| Domenico Corrado | 44 | 10120 | University of Padua | Italy |
| William J McKenna | 43 | 11525 | University College London | UK |
| Andrea Nava | 40 | 8284 | University of Padua | Italy |
| Barbara Bauce | 39 | 7611 | University of Padua | Italy |
| Harikrishna Tandri | 39 | 7747 | The Johns Hopkins University | USA |
| Daniel P Judge | 38 | 5681 | Medical University of South Carolina | USA |
| Cynthia A James | 34 | 3871 | The Johns Hopkins University | USA |

**Supplement Figure-2 The top 10 scholars with most scientific publications on ARVC since 2012**

**
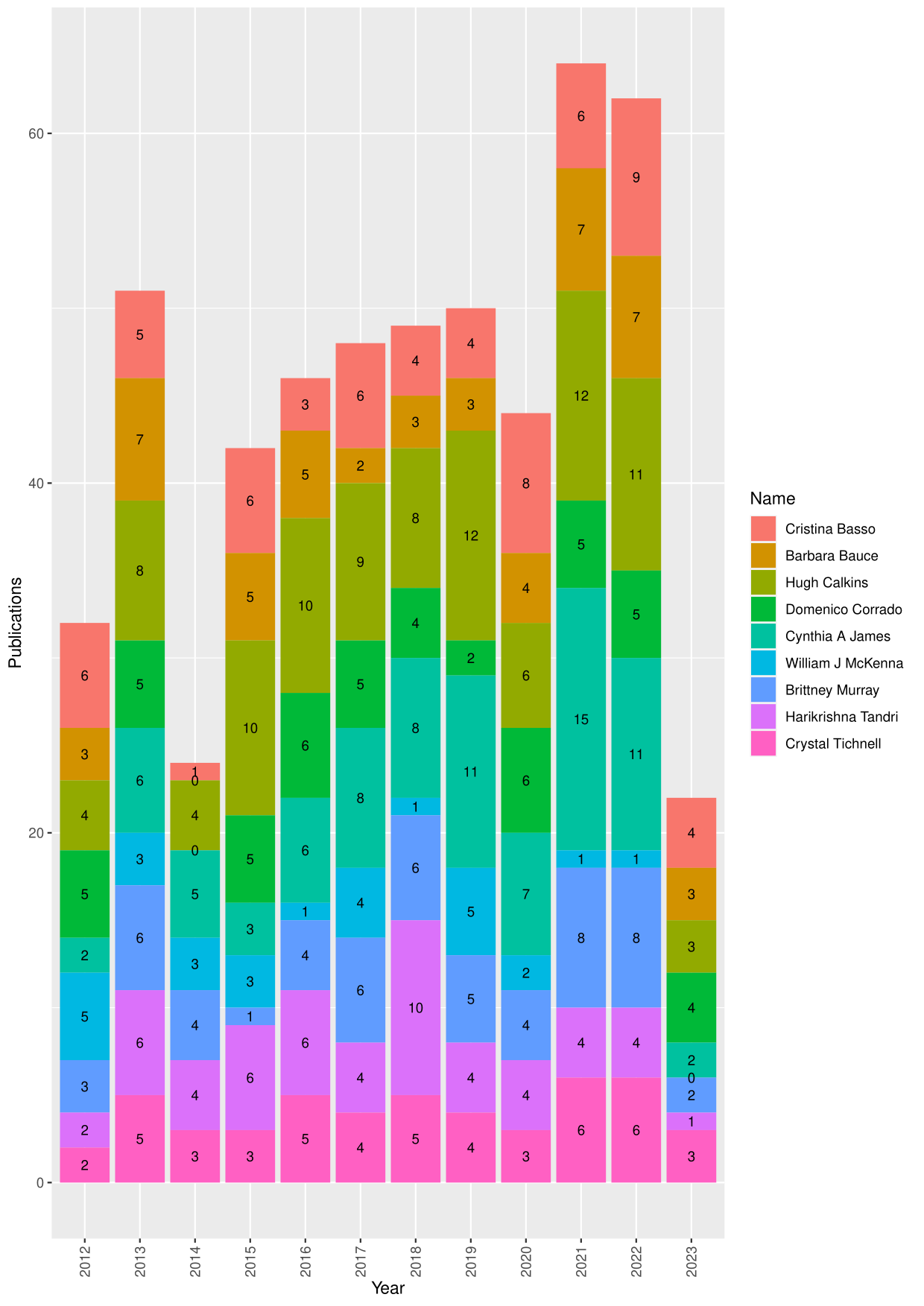
**

**Supplement table-2 The top 10 journals with most publications on ARVC**

| Journal | Article | IF (2023) | JCR (2023) | Publisher |
| --- | --- | --- | --- | --- |
| Heart Rhythm | 99 | 5.5 | Q2 | Elsevier |
| Circulation | 92 | 37.8 | Q1 | Lippincott Williams and Wilkins |
| EUROPACE | 86 | 6.1 | Q1 | Oxford University Press |
| International Journal of Cardiology | 84 | 3.5 | Q2 | Elsevier |
| Journal of Cardiovascular Electrophysiology | 82 | 2.7 | Q3 | Wiley |
| Journal of the American College of Cardiology | 76 | 24 | Q1 | Elsevier |
| American Journal of Cardiology | 75 | 2.8 | Q3 | Elsevier |
| European Heart Journal | 74 | 39.3 | Q1 | Oxford University Press |
| Pace-Pacing and Clinical Electrophysiology | 63 | 1.8 | Q4 | Wiley |
| Circulation-Arrhythmia and Electrophysiology | 52 | 8.4 | Q1 | Lippincott Williams and Wilkins |

Note: IF= Impact factor; JCR= Journal Citation Reports (Clarivate, 2023)
